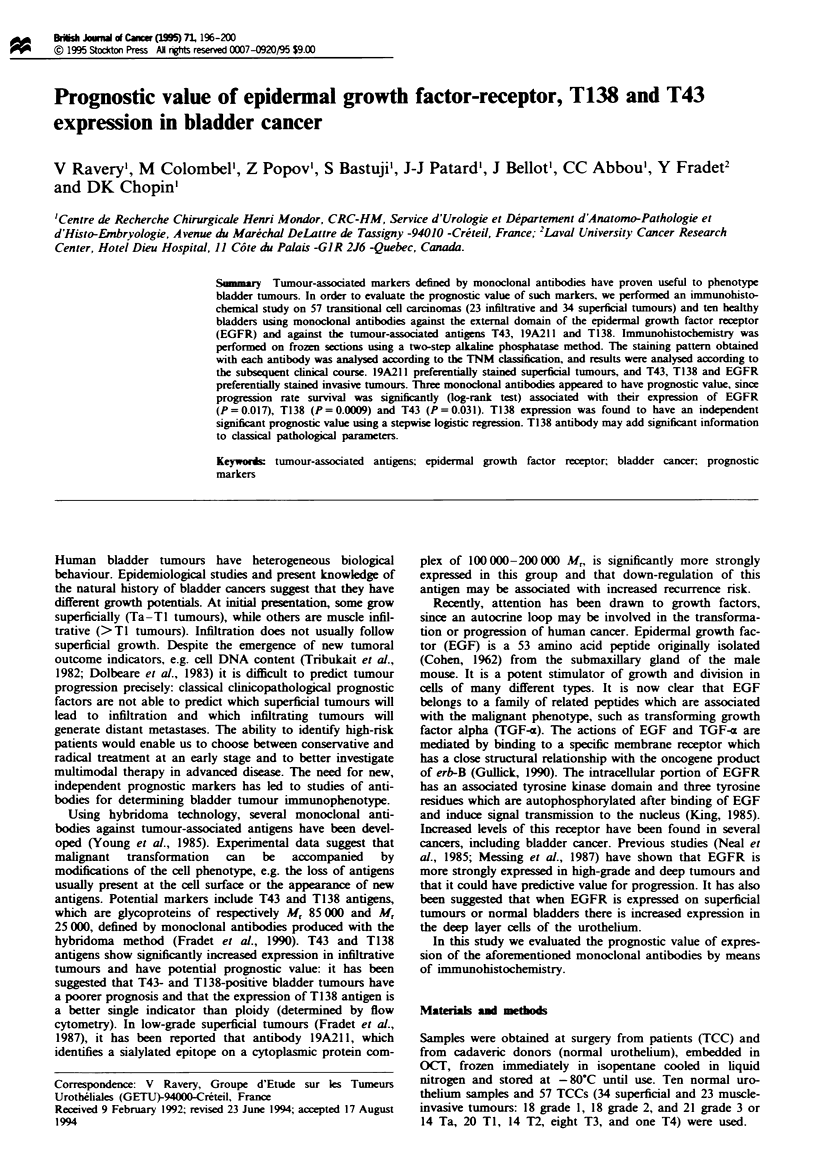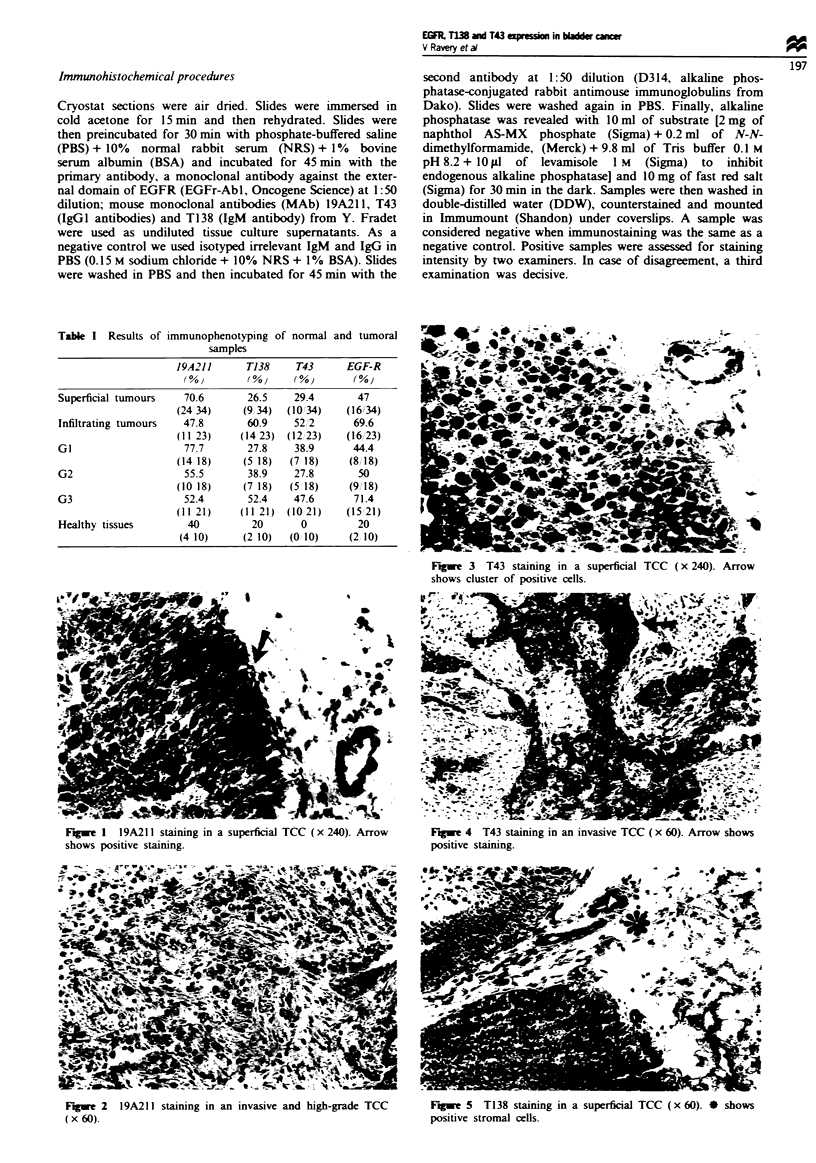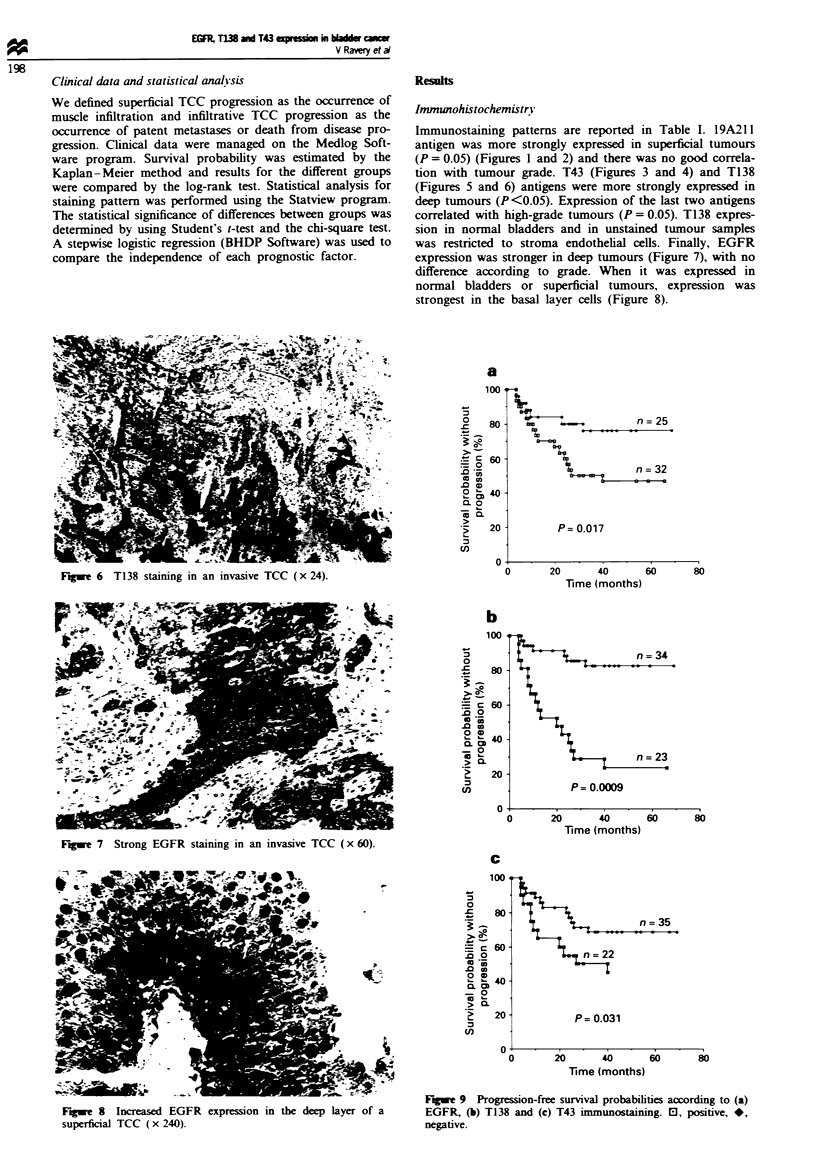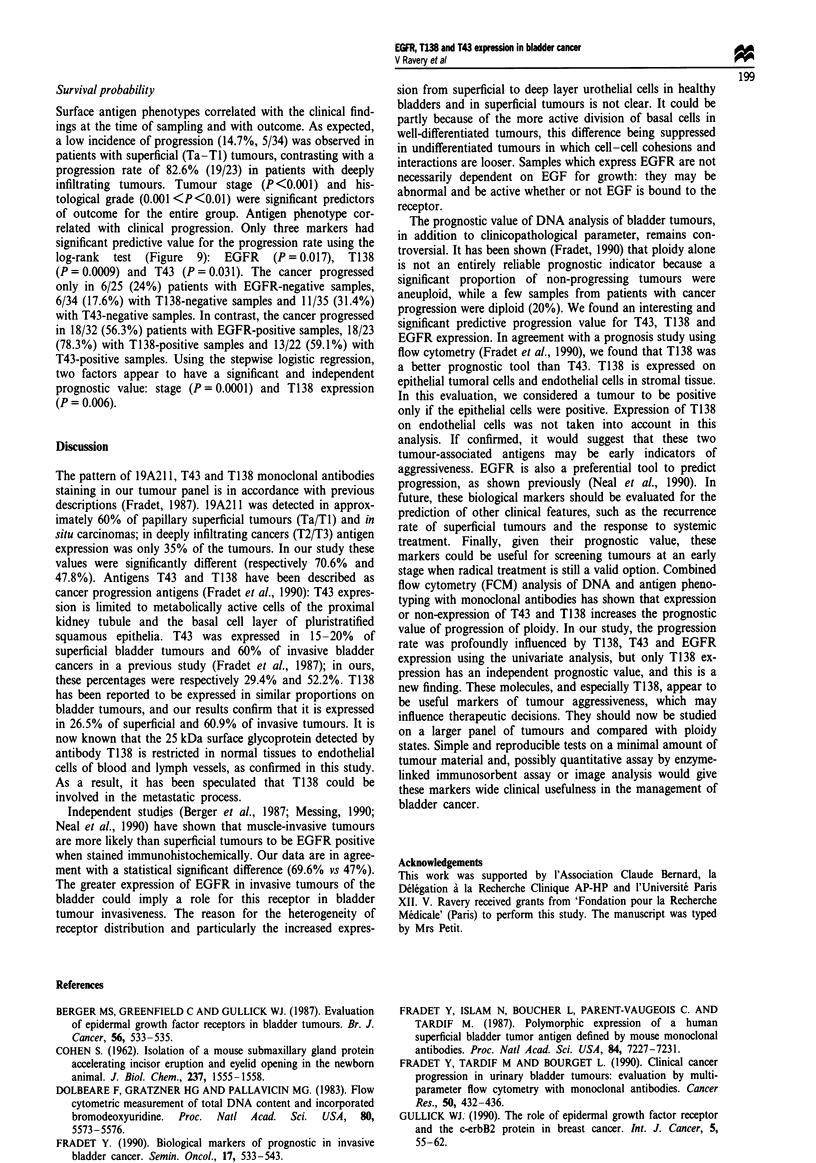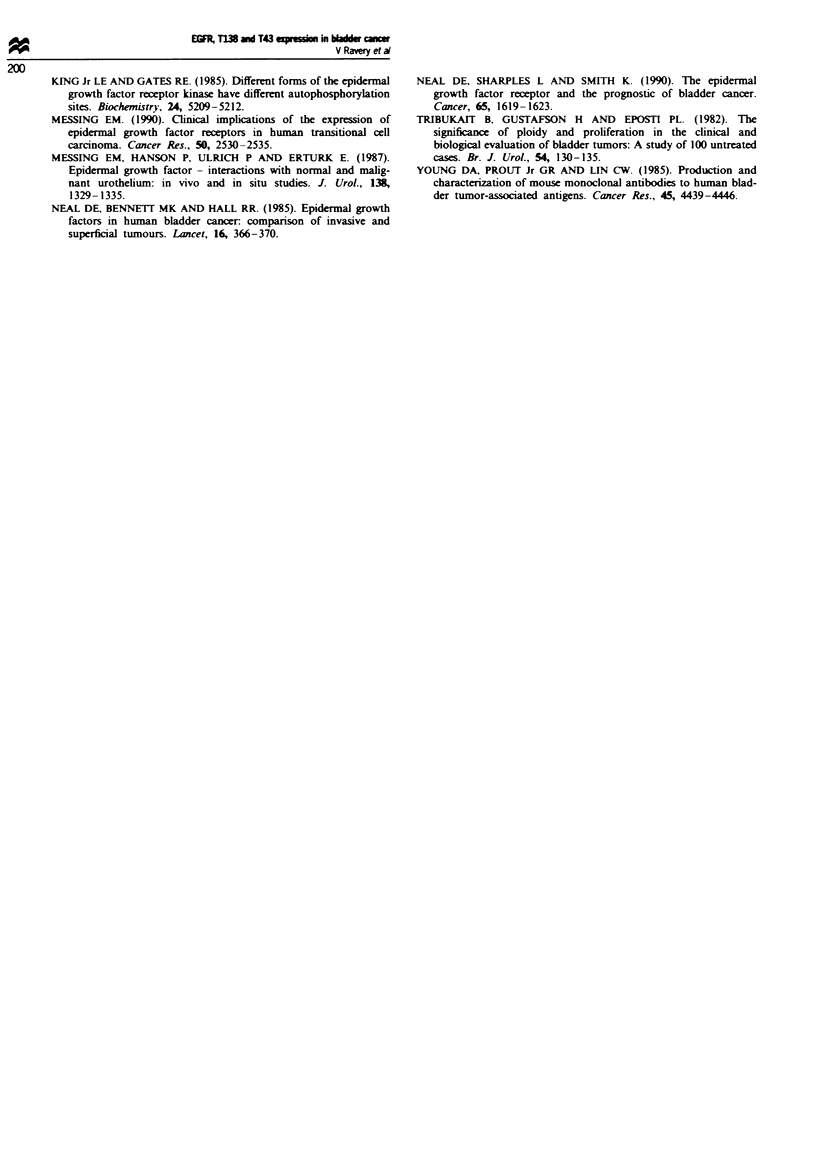# Prognostic value of epidermal growth factor-receptor, T138 and T43 expression in bladder cancer.

**DOI:** 10.1038/bjc.1995.40

**Published:** 1995-01

**Authors:** V. Ravery, M. Colombel, Z. Popov, S. Bastuji, J. J. Patard, J. Bellot, C. C. Abbou, Y. Fradet, D. K. Chopin

**Affiliations:** Centre de Recherche Chirurgicale Henri Mondor, CRC-HM, Créteil, France.

## Abstract

**Images:**


© 1995 Stockton Press All rights reserved 0007-0920/95 $9.00

### Prognostic value of epidermal growth factor-receptor, T138 and T43 expression in bladder cancer

V Ravery[1], M Colombel[1], Z Popov[1], S Bastuji[1], J-J Patard[1], J Bellot[1], CC Abbou[1], Y Fradet[2] and DK Chopin[1]

[1]*Centre de Recherche Chirurgicale Henri Mondor, CRC-HM, Service d'Urologie et Département d'Anatomo-Pathologie et d'Histo-Embryologie, Avenue du Maréchal DeLattre de Tassigny -94010 -Créteil, France;* [2]*Laval University Cancer Research Center, Hotel Dieu Hospital, 11 Côte du Palais -G1R 2J6 -Quebec, Canada.*

**Summary** Tumour-associated markers defined by monoclonal antibodies have proven useful to phenotype bladder tumours. In order to evaluate the prognostic value of such markers, we performed an immunohistochemical study on 57 transitional cell carcinomas (23 infiltrative and 34 superficial tumours) and ten healthy bladders using monoclonal antibodies against the external domain of the epidermal growth factor receptor (EGFR) and against the tumour-associated antigens T43, 19A211 and T138. Immunohistochemistry was performed on frozen sections using a two-step alkaline phosphatase method. The staining pattern obtained with each antibody was analysed according to the TNM classification, and results were analysed according to the subsequent clinical course. 19A211 preferentially stained superficial tumours, and T43, T138 and EGFR preferentially stained invasive tumours. Three monoclonal antibodies appeared to have prognostic value, since progression rate survival was significantly (log-rank test) associated with their expression of EGFR ($P = 0.017$), T138 ($P = 0.0009$) and T43 ($P = 0.031$). T138 expression was found to have an independent significant prognostic value using a stepwise logistic regression. T138 antibody may add significant information to classical pathological parameters.

**Keywords:** tumour-associated antigens; epidermal growth factor receptor; bladder cancer; prognostic markers

Human bladder tumours have heterogeneous biological behaviour. Epidemiological studies and present knowledge of the natural history of bladder cancers suggest that they have different growth potentials. At initial presentation, some grow superficially (Ta–T1 tumours), while others are muscle infiltrative (>T1 tumours). Infiltration does not usually follow superficial growth. Despite the emergence of new tumoral outcome indicators, e.g. cell DNA content (Tribukait *et al.*, 1982; Dolbeare *et al.*, 1983) it is difficult to predict tumour progression precisely: classical clinicopathological prognostic factors are not able to predict which superficial tumours will lead to infiltration and which infiltrating tumours will generate distant metastases. The ability to identify high-risk patients would enable us to choose between conservative and radical treatment at an early stage and to better investigate multimodal therapy in advanced disease. The need for new, independent prognostic markers has led to studies of antibodies for determining bladder tumour immunophenotype.

Using hybridoma technology, several monoclonal antibodies against tumour-associated antigens have been developed (Young *et al.*, 1985). Experimental data suggest that malignant transformation can be accompanied by modifications of the cell phenotype, e.g. the loss of antigens usually present at the cell surface or the appearance of new antigens. Potential markers include T43 and T138 antigens, which are glycoproteins of respectively $M_r$ 85 000 and $M_r$ 25 000, defined by monoclonal antibodies produced with the hybridoma method (Fradet *et al.*, 1990). T43 and T138 antigens show significantly increased expression in infiltrative tumours and have potential prognostic value: it has been suggested that T43- and T138-positive bladder tumours have a poorer prognosis and that the expression of T138 antigen is a better single indicator than ploidy (determined by flow cytometry). In low-grade superficial tumours (Fradet *et al.*, 1987), it has been reported that antibody 19A211, which identifies a sialylated epitope on a cytoplasmic protein complex of 100 000–200 000 $M_r$, is significantly more strongly expressed in this group and that down-regulation of this antigen may be associated with increased recurrence risk.

Recently, attention has been drawn to growth factors, since an autocrine loop may be involved in the transformation or progression of human cancer. Epidermal growth factor (EGF) is a 53 amino acid peptide originally isolated (Cohen, 1962) from the submaxillary gland of the male mouse. It is a potent stimulator of growth and division in cells of many different types. It is now clear that EGF belongs to a family of related peptides which are associated with the malignant phenotype, such as transforming growth factor alpha (TGF-α). The actions of EGF and TGF-α are mediated by binding to a specific membrane receptor which has a close structural relationship with the oncogene product of *erb*-B (Gullick, 1990). The intracellular portion of EGFR has an associated tyrosine kinase domain and three tyrosine residues which are autophosphorylated after binding of EGF and induce signal transmission to the nucleus (King, 1985). Increased levels of this receptor have been found in several cancers, including bladder cancer. Previous studies (Neal *et al.*, 1985; Messing *et al.*, 1987) have shown that EGFR is more strongly expressed in high-grade and deep tumours and that it could have predictive value for progression. It has also been suggested that when EGFR is expressed on superficial tumours or normal bladders there is increased expression in the deep layer cells of the urothelium.

In this study we evaluated the prognostic value of expression of the aforementioned monoclonal antibodies by means of immunohistochemistry.

### Materials and methods

Samples were obtained at surgery from patients (TCC) and from cadaveric donors (normal urothelium), embedded in OCT, frozen immediately in isopentane cooled in liquid nitrogen and stored at −80°C until use. Ten normal urothelium samples and 57 TCCs (34 superficial and 23 muscle-invasive tumours: 18 grade 1, 18 grade 2, and 21 grade 3 or 14 Ta, 20 T1, 14 T2, eight T3, and one T4) were used.

Correspondence: V Ravery, Groupe d'Etude sur les Tumeurs Urothéliales (GETU)-94000-Créteil, France
Received 9 February 1992; revised 23 June 1994; accepted 17 August 1994

*Immunohistochemical procedures*

Cryostat sections were air dried. Slides were immersed in cold acetone for 15 min and then rehydrated. Slides were then preincubated for 30 min with phosphate-buffered saline (PBS) + 10% normal rabbit serum (NRS) + 1% bovine serum albumin (BSA) and incubated for 45 min with the primary antibody, a monoclonal antibody against the external domain of EGFR (EGFr-Ab1, Oncogene Science) at 1:50 dilution; mouse monoclonal antibodies (MAb) 19A211, T43 (IgG1 antibodies) and T138 (IgM antibody) from Y. Fradet were used as undiluted tissue culture supernatants. As a negative control we used isotyped irrelevant IgM and IgG in PBS (0.15 M sodium chloride + 10% NRS + 1% BSA). Slides were washed in PBS and then incubated for 45 min with the second antibody at 1:50 dilution (D314, alkaline phosphatase-conjugated rabbit antimouse immunoglobulins from Dako). Slides were washed again in PBS. Finally, alkaline phosphatase was revealed with 10 ml of substrate [2 mg of naphthol AS-MX phosphate (Sigma) + 0.2 ml of *N-N*-dimethylformamide, (Merck) + 9.8 ml of Tris buffer 0.1 M pH 8.2 + 10 μl of levamisole 1 M (Sigma) to inhibit endogenous alkaline phosphatase] and 10 mg of fast red salt (Sigma) for 30 min in the dark. Samples were then washed in double-distilled water (DDW), counterstained and mounted in Immumount (Shandon) under coverslips. A sample was considered negative when immunostaining was the same as a negative control. Positive samples were assessed for staining intensity by two examiners. In case of disagreement, a third examination was decisive.

**Table I** Results of immunophenotyping of normal and tumoral samples

| | *19A211* (%) | *T138* (%) | *T43* (%) | *EGF-R* (%) |
|---|---|---|---|---|
| Superficial tumours | 70.6 (24/34) | 26.5 (9/34) | 29.4 (10/34) | 47 (16/34) |
| Infiltrating tumours | 47.8 (11/23) | 60.9 (14/23) | 52/2 (12/23) | 69.6 (16/23) |
| G1 | 77.7 (14/18) | 27.8 (5/18) | 38.9 (7/18) | 44.4 (8/18) |
| G2 | 55.5 (10/18) | 38.9 (7/18) | 27.8 (5/18) | 50 (9/18) |
| G3 | 52.4 (11/21) | 52.4 (11/21) | 47.6 (10/21) | 71.4 (15/21) |
| Healthy tissues | 40 (4/10) | 20 (2/10) | 0 (0/10) | 20 (2/10) |

**Figure 1** 19A211 staining in a superficial TCC (× 240). Arrow shows positive staining.

**Figure 2** 19A211 staining in an invasive and high-grade TCC (× 60).

**Figure 3** T43 staining in a superficial TCC (× 240). Arrow shows cluster of positive cells.

**Figure 4** T43 staining in an invasive TCC (× 60). Arrow shows positive staining.

**Figure 5** T138 staining in a superficial TCC (× 60). * shows positive stromal cells.

*Clinical data and statistical analysis*

We defined superficial TCC progression as the occurrence of muscle infiltration and infiltrative TCC progression as the occurrence of patent metastases or death from disease progression. Clinical data were managed on the Medlog Software program. Survival probability was estimated by the Kaplan-Meier method and results for the different groups were compared by the log-rank test. Statistical analysis for staining pattern was performed using the Statview program. The statistical significance of differences between groups was determined by using Student's *t*-test and the chi-square test. A stepwise logistic regression (BHDP Software) was used to compare the independence of each prognostic factor.

### Results

*Immunohistochemistry*

Immunostaining patterns are reported in Table I. 19A211 antigen was more strongly expressed in superficial tumours ($P = 0.05$) (Figures 1 and 2) and there was no good correlation with tumour grade. T43 (Figures 3 and 4) and T138 (Figures 5 and 6) antigens were more strongly expressed in deep tumours ($P<0.05$). Expression of the last two antigens correlated with high-grade tumours ($P = 0.05$). T138 expression in normal bladders and in unstained tumour samples was restricted to stroma endothelial cells. Finally, EGFR expression was stronger in deep tumours (Figure 7), with no difference according to grade. When it was expressed in normal bladders or superficial tumours, expression was strongest in the basal layer cells (Figure 8).

**Figure 6** T138 staining in an invasive TCC (× 24).

**Figure 7** Strong EGFR staining in an invasive TCC (× 60).

**Figure 8** Increased EGFR expression in the deep layer of a superficial TCC (× 240).

**Figure 9** Progression-free survival probabilities according to (**a**) EGFR, (**b**) T138 and (**c**) T43 immunostaining. □, positive, ◆, negative.

*Survival probability*

Surface antigen phenotypes correlated with the clinical findings at the time of sampling and with outcome. As expected, a low incidence of progression (14.7%, 5/34) was observed in patients with superficial (Ta–T1) tumours, contrasting with a progression rate of 82.6% (19/23) in patients with deeply infiltrating tumours. Tumour stage ($P<0.001$) and histological grade ($0.001<P<0.01$) were significant predictors of outcome for the entire group. Antigen phenotype correlated with clinical progression. Only three markers had significant predictive value for the progression rate using the log-rank test (Figure 9): EGFR ($P=0.017$), T138 ($P=0.0009$) and T43 ($P=0.031$). The cancer progressed only in 6/25 (24%) patients with EGFR-negative samples, 6/34 (17.6%) with T138-negative samples and 11/35 (31.4%) with T43-negative samples. In contrast, the cancer progressed in 18/32 (56.3%) patients with EGFR-positive samples, 18/23 (78.3%) with T138-positive samples and 13/22 (59.1%) with T43-positive samples. Using the stepwise logistic regression, two factors appear to have a significant and independent prognostic value: stage ($P=0.0001$) and T138 expression ($P=0.006$).

### Discussion

The pattern of 19A211, T43 and T138 monoclonal antibodies staining in our tumour panel is in accordance with previous descriptions (Fradet, 1987). 19A211 was detected in approximately 60% of papillary superficial tumours (Ta/T1) and *in situ* carcinomas; in deeply infiltrating cancers (T2/T3) antigen expression was only 35% of the tumours. In our study these values were significantly different (respectively 70.6% and 47.8%). Antigens T43 and T138 have been described as cancer progression antigens (Fradet *et al.*, 1990): T43 expression is limited to metabolically active cells of the proximal kidney tubule and the basal cell layer of pluristratified squamous epithelia. T43 was expressed in 15–20% of superficial bladder tumours and 60% of invasive bladder cancers in a previous study (Fradet *et al.*, 1987); in ours, these percentages were respectively 29.4% and 52.2%. T138 has been reported to be expressed in similar proportions on bladder tumours, and our results confirm that it is expressed in 26.5% of superficial and 60.9% of invasive tumours. It is now known that the 25 kDa surface glycoprotein detected by antibody T138 is restricted in normal tissues to endothelial cells of blood and lymph vessels, as confirmed in this study. As a result, it has been speculated that T138 could be involved in the metastatic process.

Independent studies (Berger *et al.*, 1987; Messing, 1990; Neal *et al.*, 1990) have shown that muscle-invasive tumours are more likely than superficial tumours to be EGFR positive when stained immunohistochemically. Our data are in agreement with a statistical significant difference (69.6% *vs* 47%). The greater expression of EGFR in invasive tumours of the bladder could imply a role for this receptor in bladder tumour invasiveness. The reason for the heterogeneity of receptor distribution and particularly the increased expression from superficial to deep layer urothelial cells in healthy bladders and in superficial tumours is not clear. It could be partly because of the more active division of basal cells in well-differentiated tumours, this difference being suppressed in undifferentiated tumours in which cell–cell cohesions and interactions are looser. Samples which express EGFR are not necessarily dependent on EGF for growth: they may be abnormal and be active whether or not EGF is bound to the receptor.

The prognostic value of DNA analysis of bladder tumours, in addition to clinicopathological parameter, remains controversial. It has been shown (Fradet, 1990) that ploidy alone is not an entirely reliable prognostic indicator because a significant proportion of non-progressing tumours were aneuploid, while a few samples from patients with cancer progression were diploid (20%). We found an interesting and significant predictive progression value for T43, T138 and EGFR expression. In agreement with a prognosis study using flow cytometry (Fradet *et al.*, 1990), we found that T138 was a better prognostic tool than T43. T138 is expressed on epithelial tumoral cells and endothelial cells in stromal tissue. In this evaluation, we considered a tumour to be positive only if the epithelial cells were positive. Expression of T138 on endothelial cells was not taken into account in this analysis. If confirmed, it would suggest that these two tumour-associated antigens may be early indicators of aggressiveness. EGFR is also a preferential tool to predict progression, as shown previously (Neal *et al.*, 1990). In future, these biological markers should be evaluated for the prediction of other clinical features, such as the recurrence rate of superficial tumours and the response to systemic treatment. Finally, given their prognostic value, these markers could be useful for screening tumours at an early stage when radical treatment is still a valid option. Combined flow cytometry (FCM) analysis of DNA and antigen phenotyping with monoclonal antibodies has shown that expression or non-expression of T43 and T138 increases the prognostic value of progression of ploidy. In our study, the progression rate was profoundly influenced by T138, T43 and EGFR expression using the univariate analysis, but only T138 expression has an independent prognostic value, and this is a new finding. These molecules, and especially T138, appear to be useful markers of tumour aggressiveness, which may influence therapeutic decisions. They should now be studied on a larger panel of tumours and compared with ploidy states. Simple and reproducible tests on a minimal amount of tumour material and, possibly quantitative assay by enzyme-linked immunosorbent assay or image analysis would give these markers wide clinical usefulness in the management of bladder cancer.

**Acknowledgements**

This work was supported by l'Association Claude Bernard, la Délégation à la Recherche Clinique AP-HP and l'Université Paris XII. V. Ravery received grants from 'Fondation pour la Recherche Médicale' (Paris) to perform this study. The manuscript was typed by Mrs Petit.

### References

BERGER MS, GREENFIELD C AND GULLICK WJ. (1987). Evaluation of epidermal growth factor receptors in bladder tumours. *Br. J. Cancer*, **56**, 533–535.

COHEN S. (1962). Isolation of a mouse submaxillary gland protein accelerating incisor eruption and eyelid opening in the newborn animal. *J. Biol. Chem.*, **237**, 1555–1558.

DOLBEARE F, GRATZNER HG AND PALLAVICIN MG. (1983). Flow cytometric measurement of total DNA content and incorporated bromodeoxyuridine. *Proc. Natl Acad. Sci. USA*, **80**, 5573–5576.

FRADET Y. (1990). Biological markers of prognostic in invasive bladder cancer. *Semin. Oncol.*, **17**, 533–543.

FRADET Y, ISLAM N, BOUCHER L, PARENT-VAUGEOIS C. AND TARDIF M. (1987). Polymorphic expression of a human superficial bladder tumor antigen defined by mouse monoclonal antibodies. *Proc. Natl Acad. Sci. USA*, **84**, 7227–7231.

FRADET Y, TARDIF M AND BOURGET L. (1990). Clinical cancer progression in urinary bladder tumours: evaluation by multiparameter flow cytometry with monoclonal antibodies. *Cancer Res.*, **50**, 432–436.

GULLICK WJ. (1990). The role of epidermal growth factor receptor and the c-erbB2 protein in breast cancer. *Int. J. Cancer*, **5**, 55–62.

KING Jr LE AND GATES RE. (1985). Different forms of the epidermal growth factor receptor kinase have different autophosphorylation sites. *Biochemistry*, **24**, 5209-5212.

MESSING EM. (1990). Clinical implications of the expression of epidermal growth factor receptors in human transitional cell carcinoma. *Cancer Res.*, **50**, 2530-2535.

MESSING EM, HANSON P, ULRICH P AND ERTURK E. (1987). Epidermal growth factor - interactions with normal and malignant urothelium: in vivo and in situ studies. *J. Urol.*, **138**, 1329-1335.

NEAL DE, BENNETT MK AND HALL RR. (1985). Epidermal growth factors in human bladder cancer: comparison of invasive and superficial tumours. *Lancet*, **16**, 366-370.

NEAL DE, SHARPLES L AND SMITH K. (1990). The epidermal growth factor receptor and the prognostic of bladder cancer. *Cancer*, **65**, 1619-1623.

TRIBUKAIT B, GUSTAFSON H AND EPOSTI PL. (1982). The significance of ploidy and proliferation in the clinical and biological evaluation of bladder tumors: A study of 100 untreated cases. *Br. J. Urol.*, **54**, 130-135.

YOUNG DA, PROUT Jr GR AND LIN CW. (1985). Production and characterization of mouse monoclonal antibodies to human bladder tumor-associated antigens. *Cancer Res.*, **45**, 4439-4446.